# Bioinspired Injectable Polyurethane Underwater Adhesive with Fast Bonding and Hemostatic Properties

**DOI:** 10.1002/advs.202308538

**Published:** 2024-02-13

**Authors:** Xiaolei Guo, Xin Zhao, Lei Yuan, Hao Ming, Zhen Li, Jiehua Li, Feng Luo, Hong Tan

**Affiliations:** ^1^ College of Polymer Science and Engineering State Key Laboratory of Polymer Materials Engineering Med‐X Center for Materials Sichuan University Chengdu 610065 China

**Keywords:** injectable, polyurethane, underwater adhesive, wound healing

## Abstract

Underwater adhesives with injectable, organic solvent‐free, strong, fast adhesion, and hemostatic properties have become an urgent need in biomedical field. Herein, a novel polyurethane underwater adhesive (PUWA) inspired by mussels is developed utilizing the rapid post‐cure reaction of isocyanate esterification without organic solvents. The PUWA is created through the injectable two component curing process of component A (biocompatible polyurethane prepolymer) and component B (dopamine modified lysine derivatives: chain extender‐LDA and crosslinker‐L_3_DA). The two‐component adhesive cures quickly and firmly underwater, with an impressive bonding strength of 40 kPa on pork skin and excellent burst pressure of 394 mmHg. Moreover, the PUWA exhibits robust adhesion strength in hostile environments with acid, alkali and saline solutions. Combined with excellent biocompatibility and hemostatic performance, the PUWA demonstrates effectively sealing wounds and promoting healing. With the ability to bond diverse substrates rapidly and strongly, the PUWA holds significant potential for application in both biomedical and industrial fields.

## Graphical abstract

This study provides a novel bio‐inspired polyurethane adhesive utilizing the rapid post‐cure reaction of isocyanate esterification without organic solvents. The two‐component adhesive cures quickly and firmly underwater in situ, exhibits robust adhesion strength. Injectable adhesive demonstrates effectively sealing wounds and promoting healing. This work provides a new option for the development of high performance injectable underwater tissue adhesives.

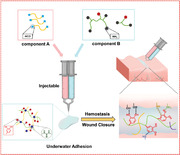

## 1 Introduction

Underwater adhesives have wide applications in various fields, including industry, marine and medical.^[^ ^1^, ^2^, ^3^ ^]^ The hydration layer of bonding interface poses a challenge to achieving strong and fast underwater adhesion.^[^ ^4^ ^]^ As an alternative to traditional surgical sutures, medical tissue adhesives are gaining popularity.^[^ ^5^, ^6^ ^]^ Nevertheless, many clinical adhesives such as cyanoacrylates and fibrinogen adhesives lose their bonding properties when exposed to water.^[^ ^7^, ^8^ ^]^ In recent years, various interface interactions including electrostatics, hydrogen bonding, covalent bonding, host–guest interaction and micro/nano‐structural modifications have been utilized to disrupt the hydration layer to enhance adhesion affinity in wet conditions.^[^ ^9^, ^10^, ^11^, ^12^ ^]^ Bioactive tissue adhesives possess adjustable biomechanical and biochemical properties that make them attractive for soft tissue wound management and wound healing.^[^ ^13^, ^14^, ^15^ ^]^ However, most of these adhesives fail to achieve true strong and rapid bonding underwater and even require complex modification processes, which limit their large‐scale and convenient application. As for medical application, it is imperative to develop an underwater medical adhesive that possesses high bonding strength, convenient injectability and biosafety.^[^ ^16^ ^]^

Various approaches to developing tough adhesives have been inspired by plants and animals.^[^ ^17^ ^]^ Marine organisms, such as mussels,^[^ ^18^ ^]^ crustaceans,^[^ ^19^ ^]^ and worms,^[^ ^20^ ^]^ secrete mucins that enable them to firmly adhere to substrates in dynamic and turbulent environments. The protein‐rich coacervate that diffuses freely, adapts to various forms, and solidifies quickly underwater, which is crucial for their attachment.^[^ ^21^ ^]^ Inspired by these organisms, post‐cure strategies have been employed to develop fluid adhesives. They can conform better to surface morphology and provide strong underwater adhesion.^[^ ^22^, ^23^ ^]^ Xu et al. utilized a solvent exchange strategy of dimethyl sulfoxide and water to construct mechanically tough adhesive, which gel formation in situ induced by solvent displacement, making it suitable as an underwater adhesive.^[^ ^24^ ^]^ Chen et al. developed a novel underwater adhesive using high dense hydrogen bonding in situ coacervation.^[^ ^25^ ^]^ This adhesive can resist water due to the strong and dense hydrogen bond complexes, which work together with hydrophobic groups, resulting in super adhesion to glass substrates. However, most existing adhesives designed with post‐curing strategies require the use of organic solvents, photothermal or other external stimulation conditions, and are unable to achieve fast‐curing adhesion.^[^ ^26^, ^27^, ^28^ ^]^ This severely limits their application in the biomedical field.

Polyurethane is a type of block copolymer composed of alternating hard and soft segments.^[^ ^29^ ^]^ Due to its exceptional biocompatibility, polyurethane has found extensive applications in the biomedical field.^[^ ^30^, ^31^, ^32^ ^]^ The polyurethane medical adhesive exhibits excellent properties such as high reactivity and room temperature curing.^[^ ^33^, ^34^ ^]^ Ideal tissue adhesives require rapid curing, whereas polyurethane prepolymers typically necessitate longer curing times, thereby limiting their use in tissue bonding.^[^ ^35^ ^]^ Urethane(urea) is formed by the reaction of isocyanate with nucleophiles such as hydroxy or amino groups, resulting in a flexible and customizable molecular structure for polyurethane.^[^ ^36^, ^37^ ^]^ The reaction rate of isocyanate with amino groups is significantly higher than that of hydroxy groups, and it can form urea group with stronger hydrogen bond to enhance the cohesive strength of the material.^[^ ^33^ ^]^ Based on the chemistry of polyurethane, selecting appropriate segmental structures, including chain extender and crosslinking agent, provide the possibility for the preparation of underwater adhesives with high bonding strength, rapid and simple application, and biosafety.

**Scheme 1:**
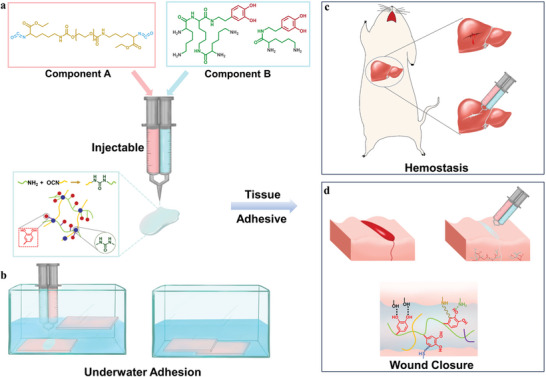
Schematic design strategy of PUWAs and their related hemostasis and wound healing applications. a) The PUWA was created through the injectable two component curing process of component A (biocompatible polyurethane prepolymer) and component B (dopamine modified lysine derivatives: chain extender‐LDA and crosslinker‐L_3_DA). b) Schematic of underwater adhesion of PUWAs. c) The PUWA was used to seal and hemostasis in rat liver wounds. d) The PUWA for wound closure and repair.

In this work, we developed mussel‐inspired polyurethane underwater adhesives (PUWAs) with rapid curing and robust adhesion for medical applications. This adhesive possessed good biocompatibility, hemostatic properties, and sufficient underwater adhesion strength for tissue seal and wound closure (**Scheme** **1**). The PUWA was created through the injectable two component curing process of component A (biocompatible polyurethane prepolymer) and component B (dopamine modified lysine derivatives: chain extender‐LDA and crosslinker‐L_3_DA). The isocyanate esterification process does not require additional stimuli or organic solvents, and can achieve strong bonding strength in as quickly as 5–10 s underwater. This adhesive is versatile and can be used on a variety of surfaces, with the catechol groups interacting with different substrates to form various physical or chemical interactions.

## 2 Results and Discussion

### 2.1 Preparation and Characterization of PUWAs

**Figure 1:**
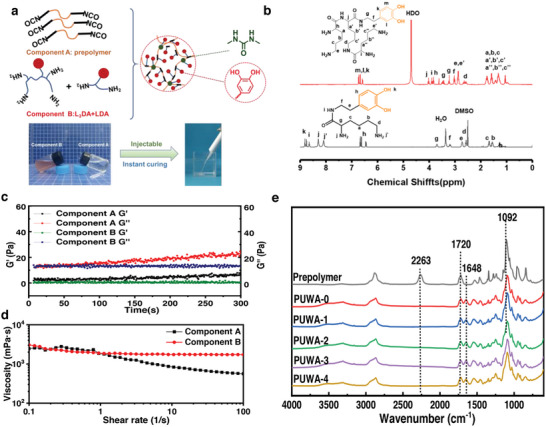
a) Design strategy of PUWAs and the photos of component A and B. b) ^1^H‐NMR spectra of LDA and L_3_DA. c) Storage modulus and loss modulus of PUWAs component A and B. d) Shear viscosity of PUWAs component A and B. e) FTIR spectra of PUWAs.

Polyurethane exhibits excellent molecular designability, making it an ideal material for preparing underwater adhesives through the rapid reaction of isocyanate and amino groups. As illustrated in **Figure** **1**a, PUWAs were synthesized by curing component A (biocompatible polyurethane prepolymer) with component B (dopamine modified lysine derivatives: chain extender‐LDA and crosslinker‐L_3_DA). We first synthesized the chain extender (LDA) and crosslinking agent (L_3_DA), with the synthesis routes and ^1^H‐NMR spectra presented in Schemes S1 and S2 (Supporting Information) and Figure 1b, respectively. The loss modulus (G″) of both components A and B exceeds the storage modulus (G'), indicating their fluid like behavior at room temperature. At shear rates below 1, components A and B exhibited similar viscosities, allowing for complete mixing (Figure 1c,d). As shown in Figure 1a, both components A and B were transparent fluids at 37 °C. The differential scanning calorimetry result of component A indicated a melting point of around 30 °C (Figure S1, Supporting Information), which allows it to transition into a liquid state below body temperature and prevents any potential burns to the skin or tissues during use. The formula of PUWAs was presented in Table S1 (Supporting Information), and five distinct adhesive formulations were created by varying the crosslinking agent content, which increased from 0 to 2.78 wt%. By injecting components A and B through a double‐barrel injection device (Figure S2, Supporting Information), rapid reaction between isocyanate group and amino group leads to stable gluing within just 10 s. Figure 1e showed the Fourier‐transform infrared (FTIR) spectra of the prepolymer and PUWAs, with a carbonyl group stretching vibration peak at 1720 cm^−1^ in the carbamate bond. In PUWAs, the absorption peak at 2263 cm^−1^ disappeared while a new peak emerged at 1648 cm^−1^ due to reaction between tetra‐amino crosslinking agent and diamino chain extender with isocyanate in prepolymer, resulting in consumption of isocyanate to form new urea group. FTIR spectra demonstrated that the prepolymer underwent rapid curing of adhesive through reaction with crosslinking agent and chain extender components.

### 2.2 Adhesive Properties of PUWAs

**Figure 2:**
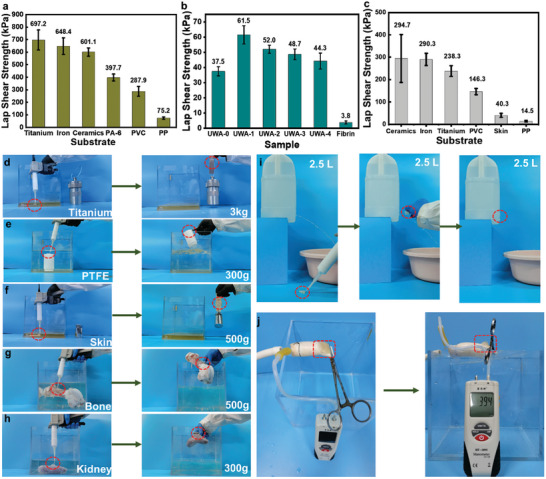
a) The lap shear strength of PUWA‐1 adhesive for different materials (including titanium, iron, ceramics, PA‐6, PVC and PP) (n = 3). b) The lap shear strength of PUWAs for pork skin (n = 3). c) The underwater adhesion of PUWA‐1 for different materials (including ceramics, iron, titanium, PVC, pork skin, and PP) (n = 3). d–h) The photos of underwater adhesion exhibition of PUWA‐1 for titanium, PTFE, pork skin, bone, and kidney (with an overlap area of about 2.5 mm × 2.5 mm). i) Photos of PUWA‐1 conducting repairs on barrel holes in a water flow environment and j) burst pressure testing of PUWA‐1 on porcine aortic tissue. The location of PUWAs were marked by red dashed box.

The adhesive does not require organic solvents and can be easily injected during use. To assess the adhesion of PUWAs, the adhesive was injected between two substrates (adhesive area: 15 mm * 25 mm) and then pressed for 5–10 s to cure quickly. Bonding tests were performed on a tensile testing machine based on a shear strength model (Figure S3, Supporting Information). As shown in **Figure** **2**a, the PUWA‐1 exhibited exceptional bonding strength with various substrates. Specifically, the PUWA‐1 exhibited adhesion strengths of 697.2±80.1, 648.4±66.2, 601.1±33.5, 397.7±28.6, 287.9±39.7, and 75.2±9 kPa on titanium, stainless steel, ceramic, polyamide (PA‐6), polyvinyl chloride (PVC), and polypropylene (PP), respectively. In addition, we evaluated the bonding strength of PUWAs to fresh pork skin. The adhesion values of PUWA‐0, PUWA‐1, PUWA‐2, PUWA‐3, and PUWA‐4 were 37.5±3.0, 61.6±6.0, 52.0±2.7, 48.7±3.5, and 44.4±5.3 kPa, respectively (Figure 2b). Notably, the adhesion of PUWAs on pork skin was more than ten times that of commercially available fibrin glue. PUWA‐0 lacked L_3_DA and exhibits poor adhesion to pork skin, while PUWA‐1 showed the strongest adhesion but PUWAs strength decreases with increasing L_3_DA content. This may be due to two reasons: first, the increase in crosslinker content leads to a decrease in total catechol content; second, the increased crosslinking speed of the adhesive may weaken its interaction force with the substrate. Catechol in adhesives can form hydrogen bond interactions with active groups on tissue surfaces. Additionally, during the adhesive application process, small molecular monomers such as LDA chain extenders and L_3_DA crosslinking agents have stronger migration abilities and can form more hydrogen bond interactions with tissue surfaces.

PUWAs can be injected and remain coacervated state in water (Movie S1, Supporting Information). When the two components were mixed, the isocyanate and amino groups reacted quickly to form a crosslinked macromolecular PUWA. The internal hydrogen bonds and crosslinking points of PUWA improve the strength and performance of the material, which can maintain the coacervated state in water during curing. By injecting PUWA‐1 underwater and applying gentle pressure for 5–10 s, it can securely adhere to various substrates such as titanium, Teflon, pork skin, bone, kidney, and others (Movie S2, Supporting Information). As illustrated in Figure 2d–h, the PUWA‐1 effortlessly withstands a load of 500 g on bone or 300 g on kidney. The underwater adhesion of PUWA‐1 to various substrates was evaluated through a shear lap test. PUWA‐1 exhibited an underwater adhesion strength of 294.7±107.1, 290.3±27.2, 238.3±24.2, 146.3±14.0, 40.3±9.0, and 14.5±3.5 kPa on titanium, 304 stainless steel, ceramics, polyvinyl chloride (PVC), pork skin, and polypropylene (PP), respectively (Figure 2c). We summarized and compared the adhesion strength of PUWA‐1 with these of currently reported adhesives in the dry and underwater states. Table S2 (Supporting Information) showed that PUWA‐1 has stronger adhesion strength to metal and pork skin than most of the bioinspired adhesives in the literatures.^[^ ^38^, ^39^, ^40^, ^41^, ^42^, ^43^, ^44^, ^45^, ^46^, ^47^, ^48^ ^]^ The rapid curing in situ and active groups of PUWAs are crucial to achieving underwater adhesion. The rapid reaction of the polyurethane two‐component underwater can realize the in‐situ contact between the adhesive and the substrate and remove water. The catechol group in PUWAs can covalently interact with ─NH_2_ and ─SH groups, form Π‐Π and cation–Π interactions with phenyl and cation, and form hydrogen bonds with ─OH, ─NH_2_, and ─COOH groups on the surface of pork skin. The ─NCO groups in PUWAs also enhance interfacial bonding by reacting with ─OH and ─NH_2_ groups on the substrate surface to form covalent bonds.^[^ ^49^ ^]^ Isocyanate can further react with water to create covalent cross‐links at the bonding interface or within the PUWAs.^[^ ^50^ ^]^ Additionally, other interactions of isocyanates with the interface such as hydrogen bonding, metal complexation, and van der Waals forces may also contribute to underwater adhesion.^[^ ^49^ ^]^ To further investigate the influence of hostile liquid environments on adhesion properties, we conducted adhesive strength tests of PUWA‐1 on titanium substrates immersed in 0.1 M HCl, 0.1 M NaOH, 4% NaCl and silicone oil solutions. As shown in Figure S4 (Supporting Information), the adhesion strengths of PUWA‐1 to titanium plates were 208.0±13.1, 126.7±5.5, 130.7±15.9, and 45.1±12.0 kPa in the respective solvents. The results indicated that the bonding strength was minimally affected by acidic environments, and partly weaken by alkaline or salinity, but significantly influenced by silicone oil environments. This could be due to the acid and alkali environment here is aqueous solution system and PUWAs in situ solidification of drainage and the ability to absorb interface water. However, the ability of PUWAs to exclude silicone oil is limited, and silicone oil is more likely to exist at the bonding interface, which will affect the effective contact between the adhesive and the substrate, so that reduce the bond strength significantly. Meanwhile, it should be noticed that the adhesion strength is still good enough for most potential applications in such hostile liquid environments, although it is certain lower than its normal underwater adhesion. Additionally, PUWA‐1 demonstrated its ability to perform underwater repairs by effectively sealing small holes in plastic buckets, unaffected by wet conditions or strong currents (Figure 2i; Movie S3, Supporting Information). To evaluate the rupture resistance of PUWA‐1, it was affixed onto the compression wound surface. Since most clinical applications only require an adhesive surface, we used PCL‐PU (a biodegradable polyurethane prepared in our laboratory) as the substrate patch as shown in Figure 2j and Movie S4 (Supporting Information). With a burst pressure of 394 mmHg, which exceeds human heart blood pressure (120 mmHg),^[^ ^7^ ^]^ PUWA‐1 demonstrated exceptional suitability for surgical procedures and hold significant clinical value.

### 2.3 Biocompatibility and Degradability Properties of the PUWAs

**Figure 3:**
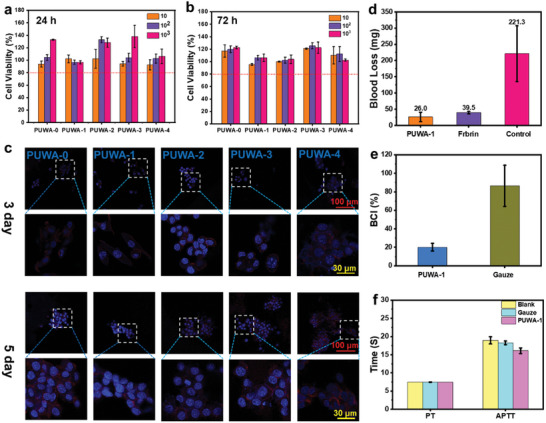
Cytotoxicity of PUWAs extracts against L929 cells at different concentrations after a) 24 and b) 72 h of incubation. c) Observation of L929 cells adhesion, and proliferation on the surface of PUWAs material. d) Blood loss of the bleeding liver treated with different strategies(n = 3). e) BCI of PUWA‐1 and gauze (n = 3). f) The impact of PUWA‐1 and gauze on APTT and PT parameters (n = 3).

Biocompatibility is an essential requirement for the application of tissue adhesives. The cytotoxicity of PUWAs extracts on L929 cells was assessed using the CCK‐8 method. Results from **Figure** **3**a,b demonstrated that the viability of L929 cells remained above 80% after 1 and 3 days. Furthermore, we investigated the attachment and proliferation of L929 cells on the surface of PUWAs. The cytoskeleton and nucleus were stained with rhodamine phalloidin (red) and DAPI (blue), respectively. Figure 3c demonstrated the culture of L929 cells on PUWAs (including PUWA‐0, PUWA‐1, PUWA‐2, PUWA‐3, and PUWA‐4). Following a 3 days incubation period, some cells adhered to the surface of the materials. Subsequently, after 5 days of culture, a significantly higher number of cells adhered to the material's surface in a spindle‐shaped pattern, indicating that PUWAs promote cellular proliferation on their surfaces. This is consistent with the reports that good biocompatibility of dopamine‐modified materials with promoting cell proliferation in literatures.^[^ ^51^, ^52^ ^]^ We also conducted hemolysis experiments on PUWA‐1, and the resulting hemolysis rate was only 1.59%, negative and positive controls were established using PBS and Triton X‐100, respectively. To further investigate the biosafety of this adhesive in vivo, we subcutaneously implanted PUWA‐1 into rats. H&E staining revealed the inflammatory behavior of surrounding tissue implanted with PUWAs, as shown in Figure S5 (Supporting Information). There were no significant alterations in the tissue sections surrounding the adhesive compared to the control group (sham operation group) from the tissue sections of day 3, 7, 14, and 28. Additionally, no tissue necrosis was detected, indicating that implantation of the adhesive did not result in a significant inflammatory response. In conclusion, results from cell experiments, hemolysis experiments and subcutaneous implantation experiments demonstrate good biocompatibility of the adhesive.

The degradation behaviors of PUWA‐1 were observed both in vitro and in vivo. In vitro degradation was performed in a simulated body fluid (SBF) containing lipase at 37 °C, while in vivo degradation was observed in a rat subcutaneous implantation model. As shown in Figure S6a (Supporting Information), PUWA‐1 showed 2–3 fold swelling both in vitro and in vivo. Over time, PUWA‐1 demonstrated on‐going weight loss. As shown in Figure S6b (Supporting Information), the initial PUWA‐1 surface was relatively smooth and flat, while after 13 days of degradation, the PUWA‐1 surface showed varying degrees of roughness and voids. After 20 days, the mass retention of PUWA‐1 was 51% and 44% in vitro and in vivo, respectively (Figure S6c, Supporting Information).

### 2.4 Hemostatic Properties of PUWAs

The excellent tissue adhesion and biocompatibility of PUWAs make it an ideal material for surgical hemostasis or wound treatment. The hemostatic efficacy of PUWA‐1 was evaluated using a mouse liver hemorrhage model (Figure 3d). In this model, the PUWA‐1 treatment group exhibited the best hemostatic effect, with significantly lower blood loss (26.0 mg) compared to the fibrin treatment group (39.5 mg) or control group (221.3 mg). This was attributed to the strong adhesion of PUWA‐1 to liver tissue, which enables it to rapidly seal wounds in the liver tissue, promote platelet aggregation at wound sites, form blood clots and accelerate hemostasis. The blood clotting index (BCI) in vitro is an important indicator of the hemostatic effect of materials, where a lower BCI indicates a better coagulation effect. Commercial gauze was used as the control material for hemostasis, and after incubating PUWA‐1 and commercial gauze with blood for 10 min at 37 °C, the BCI of gauze was found to be 86.5±22.2%, while that of PUWA‐1 was only 20.0±4.2% (Figure 3e). The excellent hemostatic performance of PUWA‐1 has piqued our interest, prompting us to investigate the possible hemostatic mechanism of PUWAs through various methods such as water absorption, PT and APTT tests, platelet adhesion and red blood cell aggregation. First, rapid water absorption is a crucial physical property of hemostatic materials and falls under the category of physical hemostasis. As shown in Figure S7 (Supporting Information), PUWAs exhibited rapid water absorption and attains equilibrium after approximately an hour, with a water uptake rate ranging from 175% to 231%. PUWAs can rapidly absorb water from blood upon contact, resulting in highly concentrated blood components. This leads to a reduction in blood flow rate, promotion of platelet aggregation, acceleration of blood coagulation and achievement of rapid hemostasis. Second, the aggregation of platelets, red blood cells and coagulation factors induced by hemostatic materials is a crucial factor affecting their efficacy. SEM images revealed that gauze had minimal adhesion of platelets and red blood cells on its surface (Figure S8a,c, Supporting Information), whereas PUWA‐1 exhibited greater accumulation of these components (Figure S8b,d, Supporting Information). Finally, the evaluation of hemostatic materials on exogenous and endogenous coagulation pathways relies heavily on prothrombin time (PT) and activated partial thrombin time (APTT) tests. As depicted in Figure 3f, both PUWA‐1 and gauze were found to reduce APTT coagulation times, but no significant difference observed in PT between PUWA‐1, gauze, and blank samples. The results indicated that PUWA‐1 possess the ability to enhance endogenous coagulation. Through animal hemostatic testing, coagulation index analysis and examination of the coagulation mechanism, it demonstrated that PUWAs exhibits excellent hemostatic performance.

### 2.5 In Vivo Wound Closure and Healing of PUWA‐1

**Figure 4:**
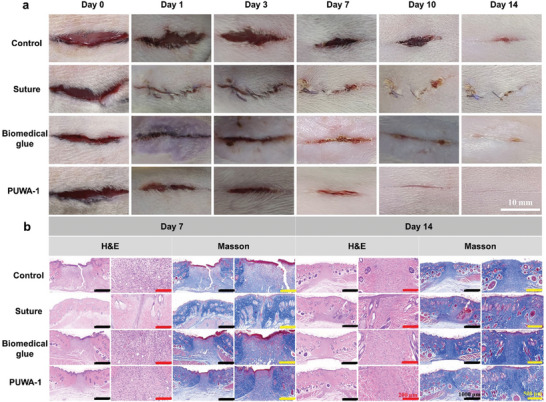
a) Images of the incisions closed by suture, biomedical glue, PUWA‐1, and the wound without treatment was set as control. b) Images of H&E staining and Masson's trichrome staining of the skin tissues after healed for 7 and 14 days (black scale bar: 1000 µm, red scale bar: 200 µm, yellow scale bar: 500 µm).

PUWAs exhibit favorable properties in terms of injectability, adhesion, biocompatibility, and hemostasis, rendering them a promising candidate for wound closure and repair. PUWA‐1 was selected to assess its efficacy in promoting wound closure and healing using a rat full‐skin incision model. Four 2 cm incisions were made on the dorsal region of anesthetized rats, and subsequently closed using sutures, biomedical glue and PUWA‐1, the untreated group served as a control. The wound healing process of each group was exhibited in **Figure** **4**a. Obviously, the incision closure effect treated by PUWA‐1, suture and biomedical glue was significantly superior to that of the untreated group; however, scar formation following suture and redness and injury caused by biomedical glue cannot be overlooked. After a duration of 14 days, the PUWA‐1 group exhibited the smallest wound surface area and significantly superior wound closure and repair effect, compared to the other groups. This can be attributed to the fact that the PUWA‐1 facilitates initial stage incision closure and promotes easier integration of new skin tissue growth.

Histological analysis was performed on days 7 and 14 to further evaluate the healing effect of the wound. Consistent with the results in Figure 4b, the incision closure effect of suture, biomedical glue and PUWA‐1 treatment was better than that of the control group. The PUWA‐1 treated wounds displayed a slight inflammatory response on day 7 due to exogenous substances, whereas the biomedical glue and control groups showed more pronounced inflammatory responses. Masson staining showed that the PUWA‐1 and suture groups had more collagen deposition, and the collagen fiber arrangement in the healed tissue was better than that in the other groups. These results showed that PUWA‐1 treated wound sites did not have infection, no serious inflammation, and the bottom of the wound fibroblasts did not have deep tissue opening. Overall, high adhesion strength and good biocompatibility of PUWA‐1 can effectively close the incision and promote sealed wound healing.

## 3 Conclusion

In summary, the injectable organic solvent‐free polyurethane adhesive exhibited rapid and extensive underwater bonding capabilities, with excellent biocompatibility and hemostatic properties that make it suitable for wound closure repair. The catechol groups in the adhesive established full contact with the substrate underwater, and after the in situ cross‐linking of component A (biocompatible polyurethane prepolymer) and component B (dopamine modified lysine derivatives: chain extender‐LDA and crosslinker‐L_3_DA), the polyurethane adhesive demonstrated robust adhesion to various surfaces. Meanwhile, it demonstrated that the polyurethane adhesive exhibited excellent biocompatibility and hemostatic performance, effectively closing wounds and promoting the healing process. The polyurethane adhesive satisfied the requirements of multifunctional tissue adhesives, featuring ease of use, absence of external stimulation, and significant potential in fields such as hemostasis, wound repair, wet bonding, and underwater repair.

## Conflict of Interest

The authors declare no conflict of interest.

## Supporting information

Supporting Information

Supplemental Movie 1

Supplemental Movie 2

Supplemental Movie 3

Supplemental Movie 4

## Data Availability

The data that support the findings of this study are available from the corresponding author upon reasonable request.
