# Supplementary material for: Bioinspired Injectable Polyurethane Underwater Adhesive with Fast Bonding and Hemostatic Properties
Source: Adv Sci (Weinh). 2024 Feb 13;11(16):2308538. doi: 10.1002/advs.202308538 (PMC11040379; doi:10.1002/advs.202308538)
Supplement: Supplementary file 1 — Supporting Information [file ADVS-11-2308538-s001.pdf]

ADVANCED SCIENCE
Open Access

Supporting Information

for *Adv. Sci.*, DOI 10.1002/advs.202308538

Bioinspired Injectable Polyurethane Underwater Adhesive with Fast Bonding and Hemostatic Properties

Xiaolei Guo, Xin Zhao, Lei Yuan, Hao Ming, Zhen Li, Jiehua Li, Feng Luo and Hong Tan**

Supporting information

Bioinspired Injectable Polyurethane Underwater Adhesive with Fast Bonding and Hemostatic Properties

Xiaolei Guo ‡, Xin Zhao ‡, Lei Yuan, Hao Ming, Zhen Li, Jiehua Li, Feng Luo*, Hong Tan*

College of Polymer Science and Engineering, State Key Laboratory of Polymer Materials Engineering, Med-X Center for Materials, Sichuan University, Chengdu 610065, China

Corresponding Author

* E-mail address: fengluo@scu.edu.cn (F.L); hongtan@scu.edu.cn (H.T).

‡ These authors contributed equally to this work.

Experimental Section

Materials. Polyethylene glycol (PEG, molecular weight 1450, Dow Chemical) was dehydrated under vacuum at 95 °C for 2 hours before use. L-Lysine diisocyanate (LDI, Nantong Dahong Chemical Industry Limited Company) was redistilled under vacuum before use. Organic bismuth (The Shepherd Chemical Company), triethanolamine (CHRON CHEMICALS), Porcine Fibrin Sealant Kit (Harnim HangBang Medical Science and Technology Co., Ltd.) and porcine skin were purchased from the commercial market. LDA (dopamine modified lysine) and L_3DA (dopamine modified lysine-tripeptide) were synthesized in our laboratory, as detail procedures presented in Supporting Information.

Synthesis of Polyurethane Prepolymer. LDI and PEG were used to synthesize polyurethane prepolymer. In sample terms, PEG (145.00 g, 0.1 mol) was added into the three-necked flask and dried under vacuum at 95 °C for 2 h. LDI (49.75 g, 0.22 mol) and two drops of organic bismuth (catalyst) were added. Polymerization was conducted for 2.5 h under mechanical stirring at 75 °C.

Preparation of Polyurethane Adhesives. The PUWAs were prepared using a two-component mixer. Component A consists of the polyurethane prepolymer, which was melted at room temperature and added to one barrel of the mixer. Component B was the dopamine modified lysine derivatives including diamino-LDA (chain extender) and tetraamino-L_3DA (crosslinking agent), dissolved in deionized water with triethanolamine (acid binding agent), which were mixed evenly and added to the other barrel. Components A and B were injected and extruded in a 2:1 ratio with a mixing glue gun before solidifying into PUWAs.

Characteristic of Polyurethane Adhesives. LDA and L_3DA were tested at 23 ±2 °C with a Bruker 400 MHz NMR spectrometer (Bruker BioSpin GmbH) using methyl sulfoxide-d6 or deuterium oxides as the solvent. Fourier-transform infrared (FTIR) spectra between 400 and 4000 cm^{-1} with a resolution of 4 cm^{-1} of PUWAs and prepolymer were obtained by Nicolet-is 50 spectrometers at room temperature. The thermal characteristics of prepolymer was acquired by DSC (DSC-250, TA Instruments, USA). The swelling tests of the PUWAs were operated by emerging the film samples with a thickness of 3 mm and a diameter of 10 mm in excess distilled water. The samples were weighed and the swelling ratios were calculated by the equation: SR = (Ws–Wd)/Wd, where Wd and Ws were the weight of the samples before and after swelling, respectively.

Adhesive Test. The normal bonding strength and underwater bonding strength of PUWAs were evaluated through shear lap test. Various substrates, including polypropylene (PP), stainless steel,

titanium, polyvinyl chloride (PVC), polyamide-6 (PA-6), ceramics and fresh pork skin were selected. Each substrate was cut into blocks measuring 75 mm x 25 mm and PUWAs were added to the substrate surface with an overlapping area of 15 mm x 25 mm. After 15 minutes, the tests were conducted using a universal testing machine with each sample repeated at least three times.The fresh porcine aorta was punctured with a 4 mm aperture, sealed using a polyurethane substrate membrane, and water was utilized as a substitute for blood. The rupture pressure was measured utilizing a digital manometer, with the procedure repeated at least three times per sample and the average value reported.

Biocompatibility of PUWAs. The cytotoxicity of PUWAs to L929 cells was determined by CCK-8 method. The PUWAs extract were diluted 10 times, 100 times and 1000 times by medium and added into 96-well culture plate inoculated with 2×10^3 cells/well, respectively. After 24 and 72 hours of culture, CCK-8 solution was added. After 4 hours, absorbance of each pore was determined by microplate reader (450 nm) to determine cell viability. The round samples of PUWAs (diameter 10 mm, thickness 1 mm) were placed at the bottom of the 24-well plate, and the cells were inoculated into the plate with the inoculation density of about 3×10^4 cells/well. After 3 and 5 days of culture, the cells were treated with 4% paraformaldehyde and 0.5% Triton solution successively, and then stained with rhodamine phalloidin and DAPI, respectively. Finally, the cell morphology was observed by laser confocal microscopy. PUWA-1 film samples(diameter 10 mm, thickness 1 mm) were subcutaneously implanted in male SD rats weighing 180 ~ 220 g to evaluate their compatibility *in vivo*. One sample was implanted on the back of each rat, and a blank control group (without implants) was included. Tissue samples were collected on days 3, 7, 14, and 28 for observation of tissue changes under an optical microscope using hematoxylin-eosin staining (H&E).

Degradation evaluation of PUWAs. To accelerate hydrolytic degradation, lipase (from Monocytogenes fluorescens) was dissolved in simulated body fluid (2g/L). PUWA-1 with a side length of 6 mm and a thickness of 1.5 mm was immersed and maintained at 37 ℃, and the degradation solution was changed every other day. PUWA-1 samples were collected at different time, and dried for dry weight measurements. PUWA-1 was implanted subcutaneously into rats, and PUWA-1 samples were collected at different time, and dried for dry weight measurement.The surface morphology of the samples after 13 days of degradation was observed by scanning electron microscopy(SEM) after freeze-drying.

Weight remaining percent(%)=$M_t/M_0\times100\%$

where M_t is the weight of sample after predetermined time, and M_0 represents the beginning weight.

Hemostatic performance of PUWA-1. The animal experiments were performed in compliance with the official guideline of the Institutional Animal Care and Use Committee of China, and carried out with the permission of the Experimental Animal Welfare and Ethics Committee of Sichuan University. The hemostatic ability of PUWA-1 *in vivo* was evaluated by rat liver bleeding model. SD rats (male, 180-220g) were anesthetized and the chest was cut open to expose the liver.A 1 cm long and 0.5 cm deep wound was created on the liver with a scalpel. The wounds were treated with PUWA-1 and commercially available fibrin adhesives respectively, and the blood sucking filter paper was weighed after 3 minutes. The blood coagulation index (BCI) was used to evaluate the coagulation effect of PUWA-1. PUWA-1 was incubated in a constant temperature water bath at 37 °C for 5 minutes, then 200 μL fresh anticoagulant rabbit blood sample was added, followed by 20 μL 0.2M calcium chloride solution and incubated at 37 °C for 5 minutes. 25 ml of deionized water was slowly added along the wall of the tube. After 10 min, the supernatant was taken and OD value at 540 nm was detected by microplate reader. Medical gauze was used as control group and anticoagulant whole blood without calcium chloride solution was used as blank group. Prothrombin time (PT) and activated partial thrombin time (APTT) are two important indexes to judge endogenous and exogenous coagulation. Platelet-free plasma (PPP, 50 μL), gauze material (control group) and PUWA-1 were mixed, incubation at 37 ℃ for 30 min, transferred to automatic coagulation analyzer (CA-5100, Sysmex, Japan), and automatically added PT reagent, APTT reagent and 0.025 mol/L calcium chloride solution according to the program. PUWA-1 and commercial gauze were placed in 24-well plates, 300 μL platelet-rich plasma was added, the samples were removed after 3 minutes of immersion, washed with PBS 3 times, platelets were fixed with 2.5% glutaraldehyde fixing solution for 1 hour, and then the samples were gradient dehydrated with ethanol aqueous solution. After drying, the surface morphology and platelet adsorption were observed by SEM. 100 μL blood drops were added to PUWA-1, incubated for 10 min, and washed 3 times with PBS to remove free red blood cells. Add 2.5% pentyl fixing solution and fix for 2 hours. The samples were dehydrated with ethanol solution and the red blood cells were observed by SEM after drying.

***In vivo* wound closure evaluation.** Six SD rats (female, 180-220g) were anesthetized and their backs were shaved and sterilized with iodine. Four incisions with length of 2 cm were made on the back of the rats, and the wounds were closed with suture, biomedical glue (Biological tissue glue,

Beijing Ji Tian Biotechnology) and PUWA-1, and the other group was a blank group. Wound images were taken on day 0, day 1, day 3, day 7, day 10 and day 14. Wound skin tissues were collected on the 7th and 14th day respectively, fixed with 4% paraformaldehyde, embedded in paraffin, and stained with hematoxylin-eosin(H&E) and Masson trichroms.

Synthesis of LDA (Lysine-Dopamine)

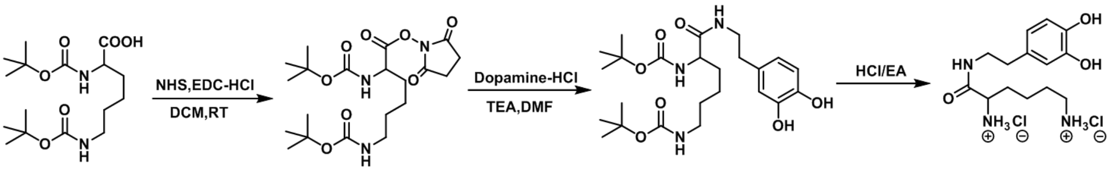

Scheme S1. The scheme of synthesis LDA.

According to the previous literature the dopamine modified lysine (LDA) was successfully synthesized in our laboratory, as shown below.[1]

(1) Synthesis of $(Boc)_2$-Lysine-NHS

The (S)-2,6-Bis-tert-butoxycarbonylaminohexanoic acid (6.93 g, 20 mmol) was dissolved in 100 mL dichloromethane, then 1-hydroxypyrrolidine-2, 5-dione (3.45 g, 30 mmol) was added into the round-bottom flask at ice salt bath. 1-Ethyl-3-(3-dimethyl aminopropyl) carbodiimide Hydrochloride (11.46 g, 60 mmol) was added into the solution being cooled to -20 °C. After 1 hour, the reaction was warmed to room temperature. After another 3 hours, TLC analysis (petroleum ether 60-90 °C /ethyl acetate 1:3) revealed the complete consumption of (S)-2, 6-Bis-tert-butoxycarbonylaminohexanoic acid. RO water was used to wash the solution 3 times. The organic layer was dried over $MgSO_4$ and removed to achieve the product.

(2) Synthesis of $(Boc)_2$-Lysine-Dopamine

The $(Boc)_2$-Lysine-NHS synthesized in the first step was dissolved in 50 mL N, N-dimethyl-Formamide. Dopamine Hydrochloride (3.79 g, 24 mmol) was added to the flask with three necks. After the Dopamine Hydrochloride was dissolved, trimethylamine (2.22 g, 22 mmol) was added to the solution. Solid was precipitated out about after 5 min reaction. The reaction proceeded for 3 h in an ice bath under a nitrogen atmosphere. After 2 hours, TLC analysis (petroleum ether 60-90 °C /ethyl acetate 1:3) showed the complete consumption of $(Boc)_2$-Lysine-NHS. The reaction mixture was quenched by the addition of dilute hydrochloric acid. RO water (200 mL) and dichloromethane (100 mL) were added to the solution and collected the underlying solution. Then, the upper layer solution was extracted with dichloromethane (80 mL×3).

The collected dichloromethane solution was washed several times with dilute hydrochloric acid. The organic layer was dried over $MgSO_4$ and removed to achieve the product.

(3) Synthesis of Lysine-Dopamine-2HCl

The $(Boc)_2$-Lysine-Dopamine synthesized in the second step was dissolved in 50 mL ethyl acetate. 40 mL HCl/ethyl acetate solution was added into a round-bottom flask, and the reaction was carried out for 3 h in an ice salt bath. Solid was precipitated out about after 20 min reaction. The precipitate was filtered and dried in under vacuum. The total yield was about 80%.

Synthesis of dopamine modified lysine-tripeptide (L_3DA)

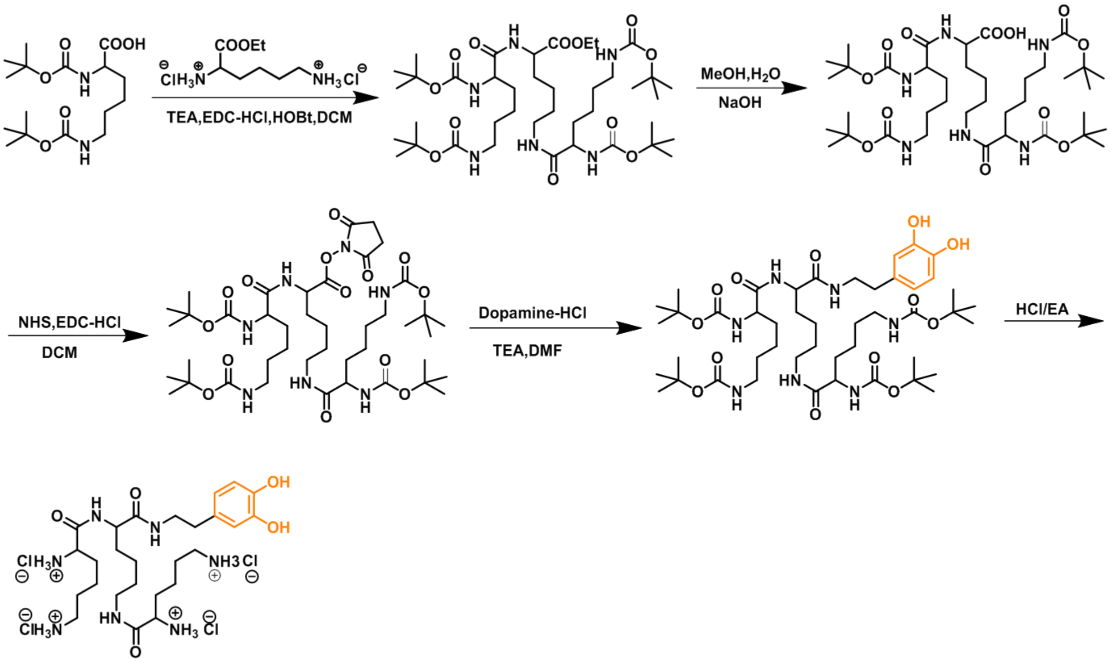

Scheme S2. The scheme of synthesis L_3DA.

(1) Synthesis of L_3-4NH-Boc-Et.

The (S)-2,6-bis-tert-butoxycarbonylaminohexanoic acid (6.228 g, 18 mmol) was dissolved in 100 mL dichloromethane, then biphenyl-4-amidoxime (3.824 g, 18 mmol), (5S)-6-ethoxy-6-oxohexane-1,5-diaminium dihydrochloride (1.483, 6 mmol), and triethylamine (6.060, 60 mmol) were added into the round-bottom flask at ice salt bath. 1-ethyl-3-(3-dimethyl aminopropyl) carbodiimide hydrochloride (3.441 g, 18 mmol) was added into the solution being cooled to -20 °C. After 1 hour, the reaction was warmed to room temperature. After another 3 hours, TLC analysis (petroleum ether 60-90 °C /ethyl acetate 1:3) revealed the complete consumption of (5S)-6-ethoxy-6-oxohexane-1,5-diaminium dihydrochloride. Saturated sodium bicarbonate solution, dilute hydrochloric acid and, RO water were used to wash the solution 3 times. The organic layer was

dried over $MgSO_4$ and removed to achieve the product. The product was purified by column chromatography (silica gel, EA：PE=1:1). $[M+Na^+]$=853.40.

(2) Synthesis of L_3-4NH-Boc-COOH.

The L_3-4NH-Boc-Et synthesized in the first step was dissolved in 100 mL methanol, then 3 M NaOH solution was added into the round-bottom flask at room temperature. After 3 hours, TLC (petroleum ether 60-90 °C /ethyl acetate 1:3) showed starting material was consumed completely. 5% dilute hydrochloric acid solution was added dropwise into the filtrate to adjust p H=8~9. And the solvent was removed under reduce pressure, then the product was dissolved in ethyl acetate, and 5% dilute hydrochloric acid was added into the filtrate. The organic phase was dried by magnesium sulfate anhydrous filtered, and the solvent was removed under reduced pressure. $[M-H^+]$=801.40.

(3) Synthesis of L_3-4NH-Boc-NHS.

The L_3-4NH-Boc-COOH synthesized in the first step was dissolved in 50 mL dichloromethane, then the flask was charged with 1-hydroxypyrrolidine-2, 5-dione and, 1-Ethyl-3-(3-dimethyl aminopropyl) carbodiimide hydrochloride under -20 °C. After 1 hour, the reaction was warmed to room temperature. After another 3 hours, TLC analysis (petroleum ether 60-90 °C /ethyl acetate 1:3) revealed the complete consumption of L_3-4NH-Boc-COOH. RO water was used to wash the solution 3 times. The organic layer was dried over $MgSO_4$ and removed to achieve the product.

(4) Synthesis of L_3-4NH-Boc-Dopa.

The L_3-4NH-Boc-NHS synthesized in the first step was dissolved in 50 mL N, N-dimethyl-formamide, and under the protection of nitrogen, dopamine hydrochloride was added into the round-bottom flask. After the dopamine hydrochloride was dissolved, trimethylamine was added to the solution. Solid was precipitated out about after 5 min reaction. After 2 hours, TLC analysis (petroleum ether 60-90 °C /ethyl acetate 1:3) showed the complete consumption of L_3-4NH-Boc-NHS. The reaction mixture was quenched by the addition of dilute hydrochloric acid. RO water (200 mL) and dichloromethane (100 mL) were added to the solution and collected the underlying solution. Then, the upper layer solution was extracted with dichloromethane (80 mL×3).
The collected dichloromethane solution was washed several times with dilute hydrochloric acid. The organic layer was dried over $MgSO_4$ and removed to achieve the product. $[M-H^+]$=936.40.

(5) Synthesis of L_3DA.

The L_3-4NH-Boc-Dopa synthesized in the second step was dissolved in 50 mL ethyl acetate. HCl/ethyl acetate solution was added into a round-bottom flask, and the reaction was carried out for 3 h in an ice salt bath. Solid was precipitated out about after 20 min reaction. The precipitate was filtered and dried in under vacuum. $[M-H^+]$=536.15.

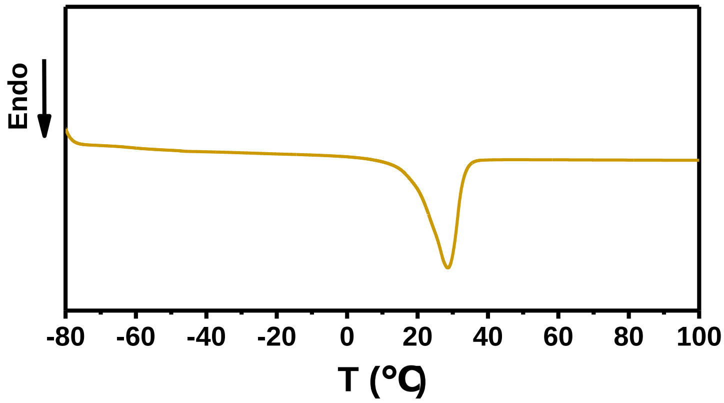

Fig. S1 DSC of polyurethane prepolymer.

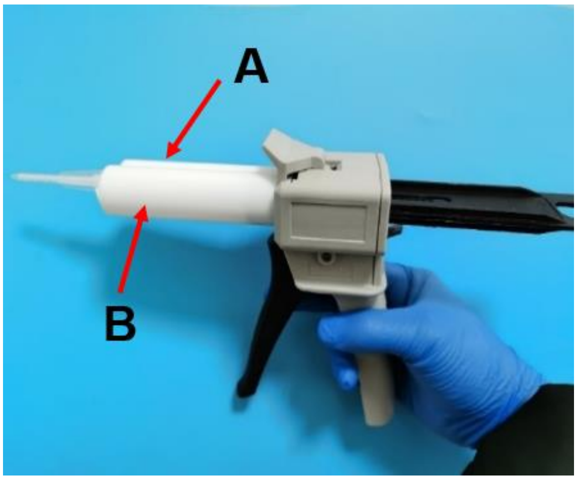

Fig. S2 The equipment of two-component mixer for preparing polyurethane underwater adhesives.

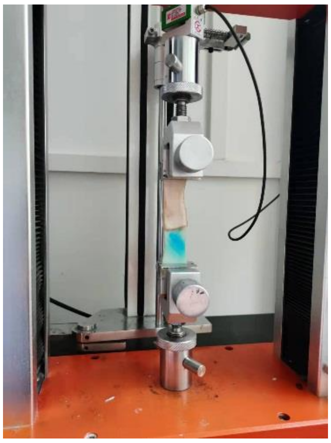

Fig. S3 The lap shear strength test of PUWAs.

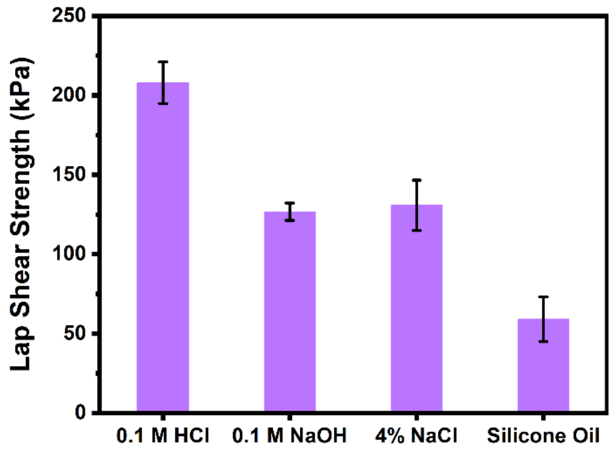

Fig. S4 The lap shear strength of PUWA-1 adhesive to titanium in different liquid environments(n=3).

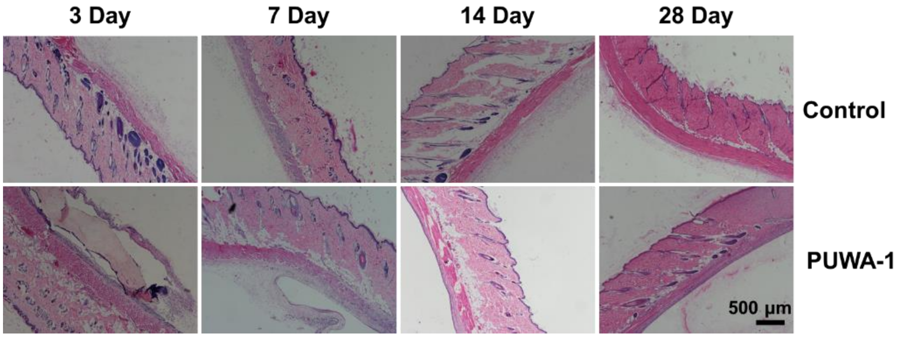

Fig. S5 Images of H&E staining of the skin tissue after subcutaneous implantation of PUWA-1 adhesive at different times.

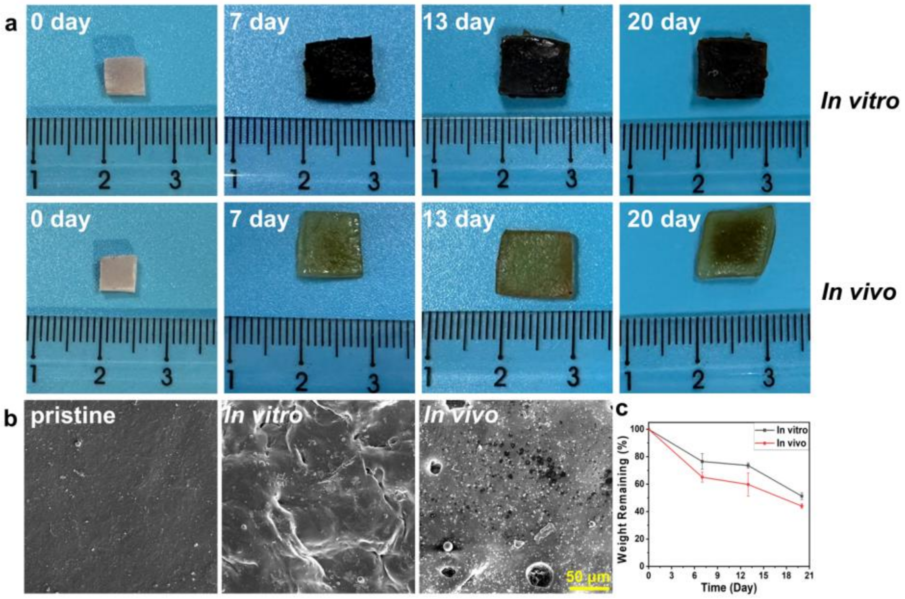

Fig. S6 (a) Images of PUWA-1 after degradation in vitro(Simulated body fluid) and in vivo(rat subcutaneous implantation) at different times, (b) SEM Images of PUWA-1 after 13 days of degradation(scale bar: 50 μm),(c) Weight remaining of PUWA-1 after degradation at different times.

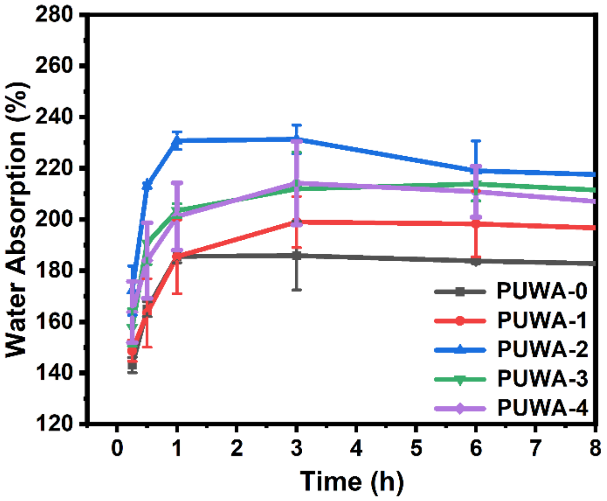

Fig. S7 Water absorption rate of PUWAs samples(n=3).

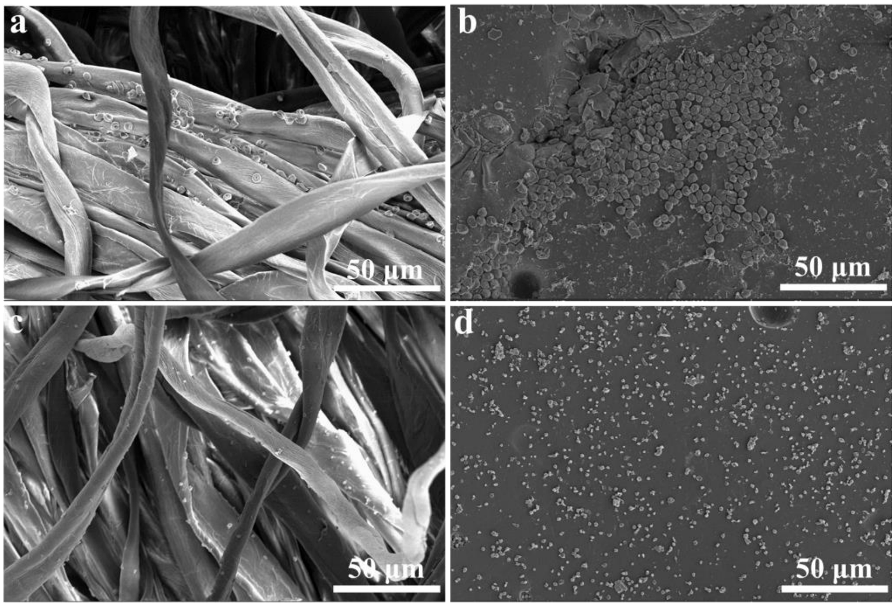

Fig. S8 (a) Aggregation state of erythrocyte on the gauze, (b) aggregation state of erythrocyte on the PUWA-1, (c) aggregation state of a platelet on the gauze, (d) aggregation state of a platelet on the PUWA-1.

Table S1. The component of PUWAs (wt%)

	PUWA-0	PUWA-1	PUWA-2	PUWA-3	PUWA-4
LDA-2HCl	15.53	15.24	14.96	13.81	12.67
L_3DA-4HCl	0.00	0.28	0.56	1.67	2.78
Prepolymer	63.28	63.29	63.29	63.32	63.34
Water	8.12	8.12	8.12	8.13	8.13
Triethanolamine	13.07	13.07	13.07	13.08	13.08

Table S2. Adhesion strength of adhesives compared to other systems reported in literatures

Adhesives	Substrate	Adhesion strength (kPa)	Condition	Ref.
Hydrogel adhesive	Metal	40	Dry	[2]
Mussel-inspired adhesive	Metal	86	Dry	[3]
Mussel-inspired adhesive	Metal	507	Dry	[4]
PUWA-1 adhesive	Metal	648	Dry	This work
Hydrogel adhesive	Metal	13	Under water	[2]
Mussel-inspired adhesive	Metal	5.4	Under water	[4]
Hyperbranched polymer adhesives	Metal	390	Under water	[5]
PVA and tannic acid adhesive	Metal	75	Under water	[6]
PUWA-1 adhesive	Metal	290	Under water	This work
Egg albumen adhesive	pork skin	56.2	Dry	[7]
Mussel-inspired adhesive	pork skin	39	Dry	[4]
Hydrogel dressing	pork skin	4.9	Dry	[8]
Bioinspired Bioadhesive	pork skin	15	Dry	[9]
PUWA-1 adhesive	pork skin	61	Dry	This work
Mussel-inspired adhesive	pork skin	34	Under water	[4]
Hydrogel adhesive	pork skin	30	Under water	[10]
Hydrogel adhesive	pork skin	60	Under water	[11]
Ctenophore-inspired hydrogel	pork skin	18.1	Under water	[12]
PUWA-1 adhesive	pork skin	40	Under water	This work

Captions of Movies:

Movie S1: The fluent injecting behavior of PUWA-1 underwater.

Movie S2: The underwater adhesive behaviors of the PUWA-1 on various materials (Titanium, Teflon, ceramics, stone, pork skin, bone, kidney, etc.)

Movie S3: The movie showing effectively sealing small holes in plastic buckets in wet conditions and strong currents.

Movie S4: The burst pressure test for the sealing porcine aorta by the PUWA-1.

Reference

[1] P. Sun, H. Lu, X. Yao, X. Tu, Z. Zheng and X. Wang, *J. Mater. Chem.*, **2012**, *22*, 10035.

[2] L. Han, M. Wang, L. O. Prieto - López, X. Deng and J. Cui, *Adv. Funct. Mater.*, **2019**, *30*, 1907064.

[3] X. Wang, Y. Si, K. Zheng, X. Guo, J. Wang and Y. Xu, *ACS Appl. Polym. Mater.*, **2019**, *1*, 2998.

[4] L. Li, H. Peng, Y. Du, H. Zheng, A. Yang, G. Lv and H. Li, *J. Mate. Chem. B*, **2022**, *10*, 1063.

[5] C. Cui, C. Fan, Y. Wu, M. Xiao, T. Wu, D. Zhang, X. Chen, B. Liu, Z. Xu, B. Qu, W. Liu, *Adv. Mater.*, **2019**, *31*, 1905761.

[6] D. Lee, H. Hwang, J.-S. Kim, J. Park, D. Youn, D. Kim, J. Hahn, M. Seo and H. Lee, *ACS Appl. Mater. Interfaces*, **2020**, *12*, 20933.

[7] K. Xu, Y. Liu, S. Bu, T. Wu, Q. Chang, G. Singh, X. Cao, C. Deng, B. Li, G. Luo and M. Xing, *Adv. Healthc. Mater.*, **2017**, *6*, 1700132.

[8] X. Zhao, H. Wu, B. Guo, R. Dong, Y. Qiu and P. X. Ma, *Biomaterials*, **2017**, *122*, 34.

[9] S. Li, N. Chen, X. Li, Y. Li, Z. Xie, Z. Ma, J. Zhao, X. Hou and X. Yuan, *Adv. Funct. Mater.*, **2020**, *30*, 2000130.

[10] Y. Chen, H. Qin, A. Mensaha, Q. Wang, F. Huang and Q. Wei, *Composites Part B*, **2021**, *222*, 109071.

[11] M. Pan, K.-C. T. Nguyen, W. Yang, X. Liu, X.-Z. Chen, P. W. Major, L. H. Le, H. Zeng, *Chem. Eng. J.*, **2022**, *434*, 134418.

[12] X. Su, Y. Luo, Z. Tian, Z. Yuan, Y. Han, R. Dong, L. Xu, Y. Feng, X. Liu and J. Huang, *Mater. Horiz.*, **2020**, *7*, 2651.
